# Novel Imaging Approaches for Glioma Classification in the Era of the World Health Organization 2021 Update: A Scoping Review

**DOI:** 10.3390/cancers16101792

**Published:** 2024-05-08

**Authors:** Vivien Richter, Ulrike Ernemann, Benjamin Bender

**Affiliations:** Department of Diagnostic and Interventional Neuroradiology, University Hospital Tübingen, 72076 Tübingen, Germany; ulrike.ernemann@med.uni-tuebingen.de (U.E.); benjamin.bender@med.uni-tuebingen.de (B.B.)

**Keywords:** glioma, machine learning, deep learning, MRI

## Abstract

**Simple Summary:**

The 2021 WHO classification of central nervous system (CNS) tumors is challenging for neuroradiologists due to the central role of the molecular profile of tumors. We performed a scoping review of recent literature to assess the existing data on the power of novel data analysis tools to predict new tumor classes by imaging. We found room for performance improvement for subgroups with lower incidence (e.g., 1p/19q codeleted or IDH1/2 mutated gliomas) and patients with rare diagnoses (e.g., pediatric gliomas, midline gliomas). More data regarding functional MRI techniques need to be collected. Studies explicitly designed to assess the generalizability of AI-aided tools for predicting molecular tumor subgroups are lacking.

**Abstract:**

The 2021 WHO classification of CNS tumors is a challenge for neuroradiologists due to the central role of the molecular profile of tumors. The potential of novel data analysis tools in neuroimaging must be harnessed to maintain its role in predicting tumor subgroups. We performed a scoping review to determine current evidence and research gaps. A comprehensive literature search was conducted regarding glioma subgroups according to the 2021 WHO classification and the use of MRI, radiomics, machine learning, and deep learning algorithms. Sixty-two original articles were included and analyzed by extracting data on the study design and results. Only 8% of the studies included pediatric patients. Low-grade gliomas and diffuse midline gliomas were represented in one-third of the research papers. Public datasets were utilized in 22% of the studies. Conventional imaging sequences prevailed; data on functional MRI (DWI, PWI, CEST, etc.) are underrepresented. Multiparametric MRI yielded the best prediction results. IDH mutation and 1p/19q codeletion status prediction remain in focus with limited data on other molecular subgroups. Reported AUC values range from 0.6 to 0.98. Studies designed to assess generalizability are scarce. Performance is worse for smaller subgroups (e.g., 1p/19q codeleted or IDH1/2 mutated gliomas). More high-quality study designs with diversity in the analyzed population and techniques are needed.

## 1. Introduction

Because the genetic profiling of brain tumors plays an increasingly prominent diagnostic role, neuroradiologists face challenges regarding the 2021 WHO categorization of central nervous system tumors [1]. Passing the previously morphological classification of brain tumors conceptually based on the presumed cell of origin (astrocytoma, oligodendroglioma, etc.), recent advances in molecular neuropathology have allowed the definition of new tumor subgroups better corresponding to brain tumor pathological etiology, more uniform disease entities and better prediction of clinical behavior and prognostics. This shift began in the 2010s with a codification in the 5th edition of the WHO classification of central nervous system tumors in 2021 (WHO 2021). With simplified terminology, molecular features now dictate classification, and joint histopathologic and molecular analysis determine tumor grade. Diffuse gliomas are primarily classified along their isocitrate dehydrogenase (IDH1/2) mutation and 1p/19q codeletion status. The definition of the O(6)-methylguanine-DNA methyltransferase (MGMT) promoter methylation status is still required for prognostication. These requirements of the previous WHO 2016 classification are now adjoined in WHO 2021 by requirements to determine the presence of CDKN2A/B homozygous deletion, EGFR amplification, and the gain or loss of chromosome 7/10 and recommendations for determining P53, TERT promoter mutation, ATRX, and DNA methylation profiles. In cases of pediatric brain tumors, the additional diagnosis of H3.K27 and MYBL1/MLBL1-alteration, H3F3A mutation, MAPK-pathway alterations, CD34 expression, or BRAF pV600E mutation are now required. While such information is essential for entity and prognostic stratification, current advances in therapeutic approaches also predict that molecular classification will allow for targeted therapeutic approaches, hopefully leading to improved clinical outcomes, as has been the case for other CNS tumors and malignancies outside of the CNS.

The role of neuroimaging in this era of molecular diagnostics is being redefined. MRI is still the workhorse for tumor detection and spatial planning of surgery and radiotherapy. However, with the prospects of more targeted therapies, non-invasive prediction of the molecular characteristics of tumors and tumor subregions is becoming more critical. Therefore, neuroradiologists must understand how novel data analytics supported by artificial intelligence, such as machine learning-based radiomics data analysis or deep learning techniques, as well as the use of novel MRI techniques such as CEST imaging or synthetic MRI, may yield new perspectives for the correlation of imaging with the molecular characteristics of brain tumors. This scoping review aims to summarize the most recent data on the utility of these approaches for classifying glioma subtypes according to the 2021 WHO CNS tumor classification system.

## 2. Materials and Methods

A scoping review was conducted to systematically map the research conducted in this area, summarize the evidence, and identify gaps in knowledge. Adult and pediatric populations were included. The research question was to determine the available data regarding the utility of novel neuroimaging techniques and data analysis approaches for the preoperative classification of glioma subtypes according to the 2021 WHO CNS tumor classification system.

### 2.1. Search Strategy

This scoping review was conducted according to the Joanna Briggs Institute methodology for scoping reviews and the reporting guidelines of Preferred Reporting Items for Systematic Reviews and Meta-Analyses Extension for Scoping Reviews (PRISMA-ScR). Quantitative, qualitative, and mixed peer-reviewed studies were included, while systematic reviews, guidelines, book sections, and editorials were excluded from the search task. The final search strategy was defined in consensus (Table 1). It included both published and unpublished primary studies in five bibliographic databases: Ovid Embase, Ovid MEDLINE, Cochrane Central Register of Controlled Trials, Web of Science, and Google Scholar, which was conducted in April 2024 with a combination of keywords and Medical Subject Heading (MeSH) terms related to the research area. Only studies in English or German were included (Table S1).

### 2.2. Study Selection and Eligibility Criteria

**Figure 1:**
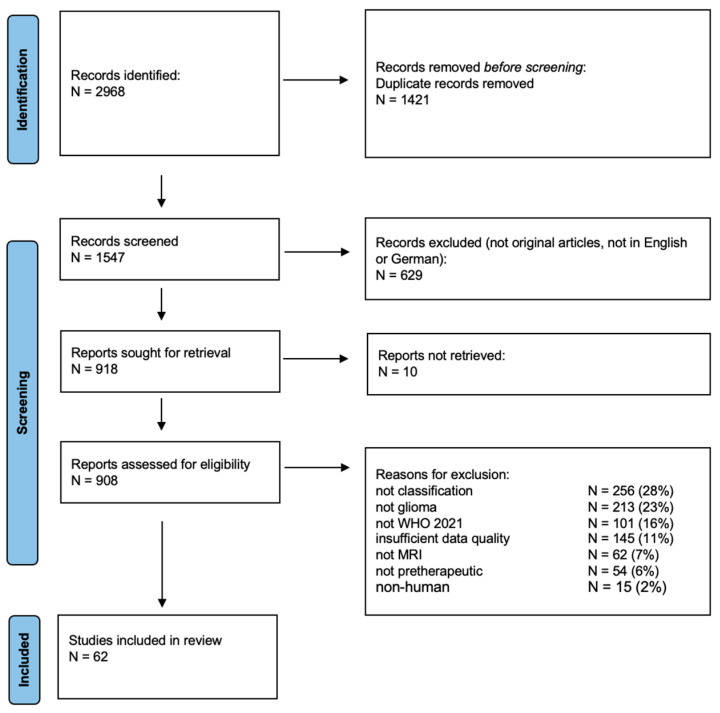
Flow diagram of study selection according to PRISMA-Scoping reviews guideline with details on exclusion criteria and the number of included and excluded studies.

All identified studies were uploaded to EndNote 20; duplicates were eliminated first by the software and then manually. Subsequently, titles and abstracts were screened by two independent reviewers to assess their eligibility. Full-text articles were retrieved, if possible, and reviewed by the same two reviewers. Reasons for exclusion related to study design and quality were charted in seven categories, as presented in Figure 1. A data-charting form was developed where data regarding bibliographic details, study design, and study results were collected. These data items were the following: the impact factor of the journal the study was published in; the source of neuroimaging data (local or public database); the number of included patients; the patient population (adult or pediatric); the analyzed tumor types; the utilized imaging sequences and the imaging sequence yielding best results; the utilized data analysis algorithm; the classification criteria (molecular subclasses); and the best AUC values. Data charting results were discussed and updated continuously in an iterative process. Any disagreements between the reviewers were resolved through discussion and consensus finding. We grouped the studies by the population, the tumor type, and the molecular subtypes and analyzed and summarized broad findings. Statistical analysis was performed in JMP^®^, Version 16.2.0, SAS Institute Inc., Cary, NC, USA, 1989–2023.

## 3. Results

### 3.1. Bibliographic Results and Eligibility Criteria

Overall, 2968 search results were reviewed, and 908 reports were retrieved in full-text version. Causes for exclusion and their proportion are shown in Figure 1. After excluding non-human studies reporting findings from animal models or cell lines (N = 15, 2%) and studies reporting CT or PET data (N = 62, 7%), 831 studies remained. Of these studies, 213 (26%) focused on non-gliomas, mainly brain metastases, meningiomas, or posterior fossa tumors, and 256 (31%) analyzed algorithms for tumor detection or segmentation and were consecutively excluded from this review. Of the remaining 362 studies, 54 (15%) focused on correlates of post-therapeutic glioma progression and pseudoprogression. Afterwards, 308 papers remained, out of which 101 (33%) were excluded due to no reference or allusion to the 2021 WHO classification despite being published in 2022 or later. Of the remaining 207 studies, 145 (75%) were excluded due to concerns related to data quality or presentation (lack of information on patient cohort, grading criteria, imaging sequences, hold-out testing cohort, or AUC results). The remaining sixty-two original high-quality articles analyzing the utility of novel MRI techniques and data analysis algorithms for the classification of neuroimaging data, according to WHO 2021, were thus included in this scoping review. The 2-year impact factor of the publishing journals ranged from 1.8 to 15.9 [2] with a mean IF of 4.5 ± 2.6 and only 17 out 62 (27%) manuscripts published in a journal with an IF above 5.

### 3.2. Patient Population

Most studies analyzed adult populations and high-grade gliomas, with only five studies (8%) analyzing pediatric populations [3,4,5,6,7], ten studies (16%) analyzing low-grade glioma [3,5,6,8,9,10,11,12,13,14] and only four studies (6%) focusing on diffuse midline glioma [15,16,17,18]. Data on the analyzed patient population are shown in Table 2. The pediatric studies included fewer patients, although the difference was not statistically significant due to the low number of pediatric studies and the large standard deviation in the adult population.

### 3.3. Data Sources

The analyzed images were mainly institution-based (imaged locally or in a multicentric setting), with only ten studies using a public cohort for external validation [7,11,19,20,21,22,23,24,25,26] and four studies using public datasets (such as the BraTs 2021 [27,28,29,30]) without including local data. The studies utilizing public datasets could include significantly more patients than those with local imaging data. These results are shown in Table 3.

### 3.4. Imaging Sequences

Most studies used conventional imaging sequences (T2, T1 with contrast agent), some with added information on visual scoring [31,32]. Only 17 studies explored parameters from diffusion-weighted imaging with one paper exploring multi-shell diffusion [33], and very few papers discussed perfusion-weighted imaging [34,35,36,37], CEST [14,15,16,17,18,19,20,21,22,23,24,25,26,27,28,29,30,31,32,33,34,35,36,37,38], or synthetic MRI [39]. The majority (73%) of studies report that combining multiple sequences is preferable. These results are shown in Table 4.

### 3.5. Molecular Subgroups

Overall, 41/62 of the reviewed studies (66%) focused on predicting IDH mutation and 1p/19q codeletion status only, while 33 studies (53%) analyzed other molecular subgroups. These were TERT [9,37,40,41,42,43,44,45,46], ATRX [8,47,48,49,50,51], H3K27 [4,15,16,17,18], MGMT [50,52,53,54,55], P53 [8,16,51,53], CDKN2A/B [12,30,35,56], EGFR [36], chr7/10 [57] and BRAF alterations [3,5,6,7]. The reported AUC values range from 0.6 to 0.98 for these predictions with an average of 0.82 to 0.9. These results are shown in Table 5.

### 3.6. Algorithms

Overall, 35 (56%) studies applied classical machine learning algorithms only (SVM, LASSO, random forest, etc.) after retrieving best-performing radiomic features from different regions of interest. In addition, 23 (37%) studies utilized deep learning methods, mainly convolutional neural networks, for radiomics-based tumor subgroup discrimination. Four studies compared multiple machine learning and deep learning algorithms. Generally, deep learning methods yielded higher AUC results for molecular classification than machine learning algorithms. The molecular subgroups where this difference was significant are listed in Table 6.

## 4. Discussion

Glioma remains one of the most challenging types of brain tumors to manage due to its heterogeneity, infiltrative nature, and variable response to treatment. With the advent of advanced molecular diagnostics in neuropathology, the visual interpretation of neuroimaging is becoming increasingly insufficient for tumor-type classification. The emergence of novel data analysis techniques presents an opportunity to redefine the role of the neuroradiologist in neurooncology. Deep learning-based methods can aid in the extraction of radiomic features and the classification of tumor subtypes. Techniques integrating multimodal data, including imaging, genomic, and clinical information, can identify complex patterns and correlations within large datasets.

Our scoping review analyzed the literature for evidence from the last two years since codifying the requirement for the molecular subtyping of brain tumors in the 2021 WHO CNS classification system. While there is a large body of written evidence on brain tumor imaging and novel MRI and data analysis techniques, thorough review and quality control dissipates a large proportion of the published studies. We found that a distinct proportion of the research body focuses on tumor detection and segmentation, which is a vital processing step preceding reporting and large-scale data analysis. However, most studies concerning tumor classification were still based on manual segmentation, which is time consuming and has lower reproducibility. We also retrieved numerous studies dealing with heterogeneous tumor entities, e.g., brain metastases or meningiomas. However, the incidence and the prognostic and therapeutic landscape of primary brain tumors warrant the focus of research in this area. In further analysis, a significant proportion of the literature published since 2022 did not allude to the 2021 WHO classification. Most of the remaining research papers showed inadequacies regarding study design, methodological reporting, and communication of results. Ultimately, only 7% of the initially retrieved manuscripts were included in this scoping review.

We found a disproportionate lack of studies about pediatric brain tumors, diffuse midline glioma, and low-grade glioma, which is explained by the lower incidence of these tumor types; however, the representation of these tumor types should be an essential element of further research.

More high-quality data on the utility of functional imaging and novel MRI techniques (e.g., CEST or MR fingerprinting) are needed. Most studies report that a combination of conventional MRI sequences yields appropriate results.

The majority of the relevant literature focuses on predicting IDH mutation and 1p/19q codeletion status, which has been required since the 2016 WHO classification. Imaging correlations for other molecular subgroups required for WHO classification since 2021 are underrepresented.

The high AUC values reported at 0.8–0.9 are mostly based on including all tumor grades and types. However, as glioblastomas are the most common adult primary malignant brain tumor, they typically make up a large portion of the dataset, and smaller subgroups usually had a worse diagnostic performance (e.g., only 38.5% of oligodendroglioma and 62.9% of IDH-mutated astrocytoma were correctly identified compared to 90.6% of IDH-wild-type glioblastoma in [19]). There is only a small number of studies with the explicit reporting of AUC of tumor subgroups or focusing on low-grade tumors.

Radiomics studies with classic machine learning algorithms prevail, but there is an increasing trend toward deep learning applications for feature extraction and subtype classification. Generally, deep learning methods yielded higher AUC results for molecular classification than machine learning algorithms. Even in the era of large public datasets, only one-fifth of the included studies utilized this data, thus limiting evidence about generalizability.

While the papers included in this scoping review were selected due to their clear methodology regarding their algorithms, there are several limitations inherent to the use of machine learning and deep learning-based medical applications. The multitude of methodologies implies limited comparability. The relatively low patient numbers and limited use of public datasets limit the diversity of the included patient populations. The study designs with relatively large training and small testing datasets and inherently imbalanced tumor subgroup populations present an inherent methodological bias, and validation in non-preselected native populations is missing. Integration into existing healthcare infrastructure and discussions on data security and ethical considerations surrounding AI-driven decision making are needed. Future research directions should focus on developing reproducible, interpretable AI models, enhancing data interoperability and standardization, leveraging multimodal data integration, and conducting prospective clinical trials to evaluate the clinical utility of AI-based tools.

A limitation of this scoping review is that the included manuscripts reflect the subjective evaluation criteria of the reviewers, resulting in an inherent bias in the selection process. The overall level of evidence of the reviewed manuscripts was Level 3 (case-control studies and retrospective comparative studies of nonconsecutive patients without consistently applying the reference “gold” standard).

Some high-quality papers with topics related to but not quite fitting could not be included in this review. Several research groups are studying imaging correlates of cellular and genetic heterogeneity in gliomas either in different regions of interest or in imaging-based tumor habitats [58,59,60,61,62,63]. A technical consideration has been raised in [64] regarding the effect of motion on classification results, suggesting a further need for research in such technical aspects. Another interesting aspect is the impact of preprocessing on the generalizability of data [25,65].

We extended the knowledge base provided by previous reviews by focusing on recent literature utilizing the 2021 WHO classification, including pediatric tumors, low-grade gliomas, and deep-learning applications. We found that radiomics data from multiparametric conventional MRI combined with deep learning applications yield high accuracy in predicting molecular markers, which is followed by classical machine learning algorithms. Despite a plentitude of tools for automated tumor detection and segmentation, these were underutilized in studies focused on tumor classification. We identified research gaps concerning pediatric tumors and lower-grade glioma subgroups as well as technical considerations regarding the quality of input data and the interpretability and generalizability of algorithms. Relevant future applications may be anticipating changes in the molecular composition of gliomas and providing information about the cellular and genetic heterogeneity of glioma subregions.

## 5. Conclusions

Research on MR-based AI-aided detection of molecular features in gliomas for neuroradiological prediction of tumor subtypes according to the 2021 WHO classification system is trending, but high-quality study designs are scarce, and generalizability is not yet achieved. Despite the excellent overall diagnostic performance of algorithmic approaches, it appears that balancing methods are underutilized, resulting in worse performance for smaller tumor subgroups (e.g., 1p/19q codeleted or IDH1/2 mutated gliomas). There is a lack of studies involving patients with rare diagnoses (e.g., pediatric gliomas, midline gliomas) and utilizing novel MRI techniques (e.g., CEST or synthetic MRI). In summary, by focusing research efforts on studies that enable generalizability, neuroradiology has the chance to retain its central diagnostic role in the era of molecular tumor classification.

## Figures and Tables

**Table 1:** Search terms applied in the literature search for this scoping review.

Topic	Search Term	Syntax
Glioma	Glioma OR brain tumor OR brain neoplasms OR tumor of the brain OR brain cancer	AND
Algorithm	radiomic* OR imaging genomic* OR radiogenomic* OR machine learning OR deep learning OR support vector machine OR artificial intelligence	AND
Imaging technique	Magnetic Resonance Imaging OR magnetic resonance tomograph OR MRI OR MR imaging OR magnetic resonance brain imaging	AND
Subjects	Animals	NOT
Date	2022/01/01:3000/12/31	AND

**Table 2:** Distribution of the analyzed patient populations in the studies included in this review. (LGG: low-grade glioma. DMG: diffuse midline glioma).

Population	N (Studies)	% (All Studies)	Number of Patients per Study	
			Mean	Stdev
Gliomas (all subtypes)	48	77%	337	445
LGG	10	16%	223	146
DMG	4	6%	141	66
adult	57	92%	317	414
pediatric	5	8%	185	118

**Table 3:** Source of imaging data per study design group and patient numbers (minimum, maximum, mean, and standard deviation) in each group. * The mean patient number was significantly higher in the studies that utilized publicly available datasets than in those using institutional data only.

Data Source	N (Studies)	% (All Studies)	Number of Patients per Study		
			Mean	Stdev	*p* *
local	48	77%	207	152	
public	4	6%	812	1225	0.001
local and public	10	16%	581	461	0.003

**Table 4:** Distribution of analyzed MRI sequences and results on the best sequences for optimal prediction results as reported by the studies included in this review (T1CE: contrast-enhanced T1w imaging. DWI: diffusion-weighted imaging. ADC: apparent diffusions coefficient. DTI: diffusion tractographic imaging. DKI: diffusion kurtosis imaging. PWI: perfusion-weighted imaging. DCE: dynamic contrast-enhanced imaging. DSC: dynamic susceptibility-weighted imaging. CEST: chemical exchange saturation. SyMRI: synthetic MRI).

Sequences	N (Studies)	% (All Studies)	Best Sequence	N	%
T2	52	84%	combination	45	73%
T1CE	48	77%	not applicable	11	17%
T2-FLAIR	36	58%	ADC	2	3%
T1	35	56%	T1CE	1	2%
DWI (ADC, DTI, DKI)	17	27%	T1CE, ADC	1	2%
PWI (DCE/DSC)	4	6%	T2	2	3%
T2*/SWI	1	2%			
CEST	2	3%			
SyMRI	1	2%			

**Table 5:** Studies with prediction efforts for different molecular subgroups relevant to the 2021 WHO classification system and their respective AUC results.

Molecular Subgroup	N of Studies	% (All Studies)	AUC			
			Min	Max	Mean AUC	Stdev
IDH1/2	28	45%	0.7	0.98	0.87	0.07
1p/19q codel	13	21%	0.6	0.98	0.84	0.11
TERT	9	15%	0.7	0.95	0.86	0.08
ATRX	6	10%	0.67	0.95	0.83	0.11
H3K27	5	8%	0.89	0.92	0.9	0.01
MGMT	5	8%	0.57	0.98	0.85	0.16
P53	4	7%	0.77	0.97	0.85	0.09
CDKN2A/B	4	7%	0.82	0.95	0.88	0.07
BRAF	4	7%	0.73	0.87	0.79	0.07
EGFR	1	2%			0.8	
chr7/10	1	2%			0.85	

**Table 6:** Molecular subgroups where a significant difference in AUC results was found between deep learning and machine learning methods.

Mean AUC	*p* (DL > ML)	*p* (DL < ML)
IDH	0.009	
TERT		0.01
ATRX	0.01	
MGMT	0.01	
CDKN2A/B	0.04	

## Supplementary Materials

The following supporting information can be downloaded at https://www.mdpi.com/article/10.3390/cancers16101792/s1, Table S1: Comprehensive list and data analysis of included manuscripts.
